# Cyclophilin D reduces Ca^2+^ sequestration by complement 1q binding protein

**DOI:** 10.1042/BCJ20253361

**Published:** 2025-12-17

**Authors:** Oluwatobi Adegbite, Yetunde Adegbite, Catrin Pickering, David N Criddle, Lu-Yun Lian

**Affiliations:** 1 Institute of Systems and Molecular Biology, Biosciences Building, University of Liverpool, Institute of Systems and Molecular Biology, Biosciences Building University of Liverpool, Liverpool L69 7ZB, U.K; 2 Department of Biochemistry, Faculty of Basic and Applied Sciences, Osun State University, Osogbo, Osun State, Nigeria

**Keywords:** C1qBP, calcium, cyclophilin D, mitochondria, permeability transition pore

## Abstract

Complement 1q binding protein (C1qBP) and cyclophilin D (CypD) are mitochondrial matrix proteins; C1qBP has been implicated in many cellular processes, including the regulation of oxidative phosphorylation, and CypD is widely associated with the regulation of mitochondrial permeability transition pore (mPTP) opening. In this study, C1qBP and CypD were shown, *in vitro*, to form a stable protein–protein complex. CypD–C1qBP interaction was disrupted by cyclosporin A and compromised by mutations of the CypD active site residues R55 and R82. AlphaFold protein modelling revealed that the large negatively charged surface of C1qBP binds to the positive surface of CypD. This electrostatically driven interaction was confirmed by the pH dependence of the protein–protein interaction, with lower affinities observed at higher pH values. C1qBP was shown to undergo conformational changes when bound to Ca^2+^ in vitro, conferring multiple Ca^2+^ interaction sites in a multi-phase process, thereby indicating that C1qBP may act as a Ca^2+^ sequester. In contrast, CypD binding to C1qBP diminished the Ca^2+^-induced conformational changes in C1qBP, lowering its Ca^2+^-binding capacity. Our findings suggest that C1qBP functions as a mitochondrial Ca^2+^ chelator, with its efficiency reduced by CypD, this most likely due to CypD and Ca^2+^ both competing for the same negative surface of C1qBP. The parallels between the features of CypD–C1qBP interaction and the regulation of Ca^2+^-dependent mPTP opening by CypD highlight a possible functional role of CypD which has so far been elusive.

## Introduction

The peptidyl prolyl *cis-trans* isomerase (PPIase) cyclophilin D (CypD) is a mitochondrial matrix protein whose biological role has mainly been associated with being an important regulator of mitochondrial permeability transition pore (mPTP). The PPIase activity of CypD is inhibited by the immunosuppressant drug cyclosporin A (CsA), an archetypal inhibitor of the mPTP. Although there is evidence that ATP synthase and the adeninine nucleotide translocator (ANT) form the principal structural components of the mPTP, the precise molecular identity of this megachannel remains uncertain [1–7]. Likewise, even though CypD has been shown to interact with the oligomycin sensitivity conferring protein (OSCP) component of the ATP synthase [8], the significance of this interaction and the mechanism by which CypD regulates the mPTP require more investigations.

Complement 1q binding protein (C1qBP), another mitochondrial protein, has also previously been proposed as a potential regulator of the mPTP [9]. Notably, C1qBP is a mitochondrial protein that has been widely implicated in many cellular processes, which principally regulate oxidative phosphorylation [10–17]. Analyses of gene expression levels of the *PPIF* gene (for CypD) and *C1QBP* (for C1qBP) genes [18] show parallel expression patterns in almost all tissues in normal and tumour states. Indeed, both *C1QBP* and *PPIF* were characterised by overall higher expression patterns in heart, intestine and adrenal tissues over other tissues analysed in a 20-week-old human foetus [19].

C1qBP may also participate in mitochondrial calcium homeostasis since its negatively charged surface could be important for metal chelation and sequestration [20–22], although to date there is no experimental evidence of C1qBP–Ca^2+^ binding. C1qBP was also shown to be overexpressed in breast, lung and colon cancer cell lines [23], suggesting a role in cell death resistance. The roles of C1qBP in the opening of the mPTP have been less defined. Whilst Chowdhury et al. [24] reported C1qBP to be an activator of pore opening based on their observations of reactive oxygen species (ROS) generation and mitochondrial dysfunction in cells which overexpressed C1qBP, McGee and Baines contend that C1qBP inhibited mPTP opening [9,23]. In the latter studies, C1qBP overexpression resulted in higher mitochondrial calcium retention capacity after ROS treatment of mouse embryonic fibroblasts MEF cells, although this was not replicable in their animal studies. However, haploinsufficiency of C1qBP sensitised isolated mouse cardiac mitochondria to Ca^2+^ induced mPTP both by mitochondria swelling and calcium retention capacity assays, an effect that involved interactions with CypD [9].

Calcium signalling in mitochondria is complex. The role of calcium chelators and calcium sequestration as a regulator of calcium-induced processes in mitochondria is not thoroughly understood. To gain an insight into regulatory mechanisms mediated via the CypD–C1qBP axis in mitochondria, including possibly the mPTP, the relationship between C1qBP and CypD and the calcium binding characteristics of C1qBP, alone and as a complex with CypD, were examined here using *in vitro* methods. The results show that C1qBP is an efficient Ca^2+^ sequester, possibly with its dynamic structure and large negative surfaces enabling it to accommodate high numbers of Ca^2+^. The presence of CypD stabilises C1QBP and appears to diminish its capacity for effective Ca^2+^-binding.

## Results

### CypD stably interacts with C1qBP

**Figure 1:**
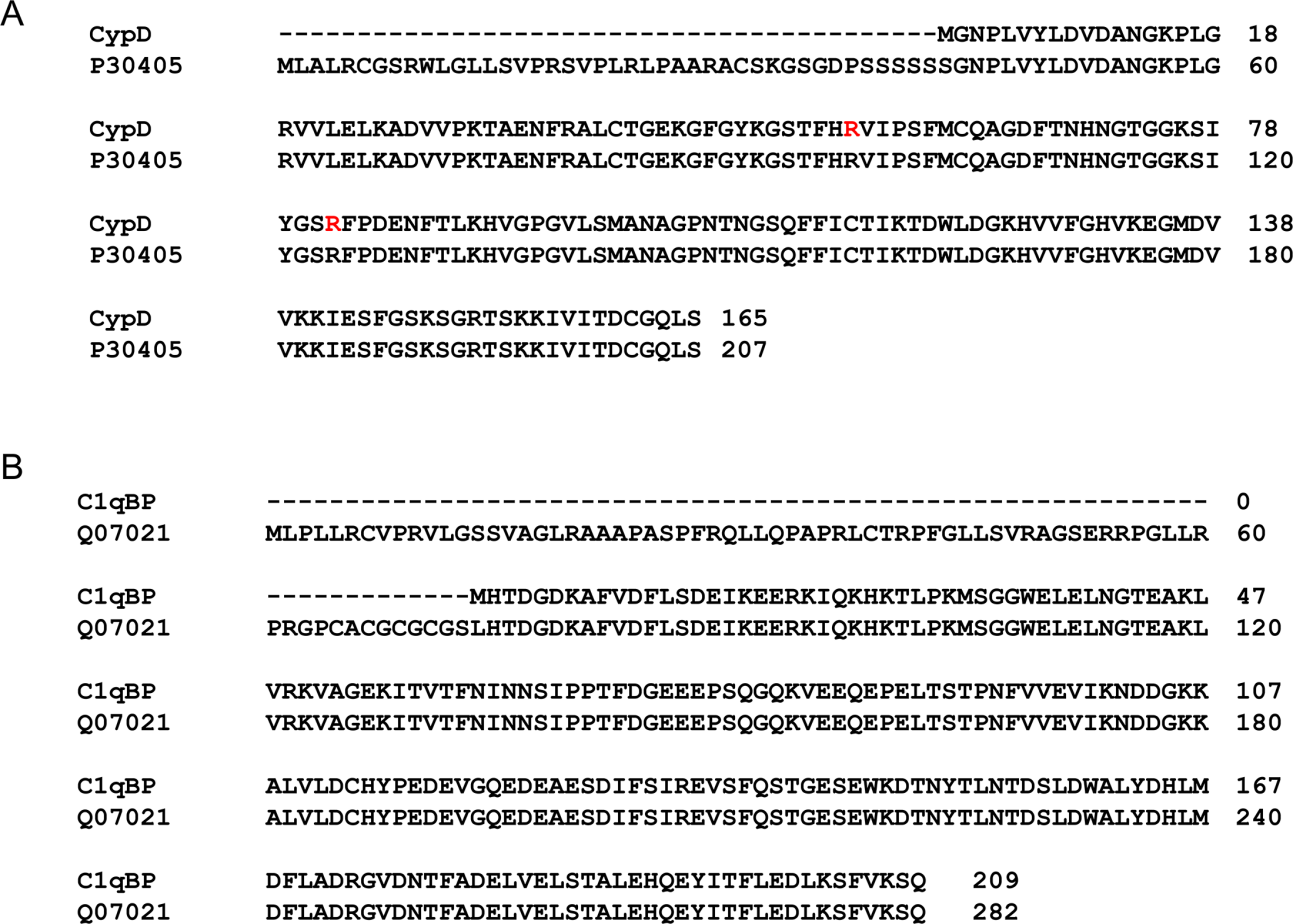
Sequences of CypD and C1qBP. (A) Sequence of the CypD, with mutations R55K and R82K highlighted in red, used in this study (upper row) aligned with the full-length protein UniProt P30405 (lower row) ( **(B)** Sequence of C1qBP used in this study (upper row) aligned with the full-length protein UniProt Q07021 (lower row). The numbering system for the truncated proteins used in the text and NMR spectra are shown

The interactions between expressed recombinant CypD and C1qBP (sequences in Figure 1) were investigated using pull-down experiments, protein stability assays, size-exclusion chromatography-multi-angle light scattering (SEC-MALS), nuclear magnetic resonance (NMR) spectroscopy, and dynamic and static light scattering (DLS/SLS).

**Figure 2:**
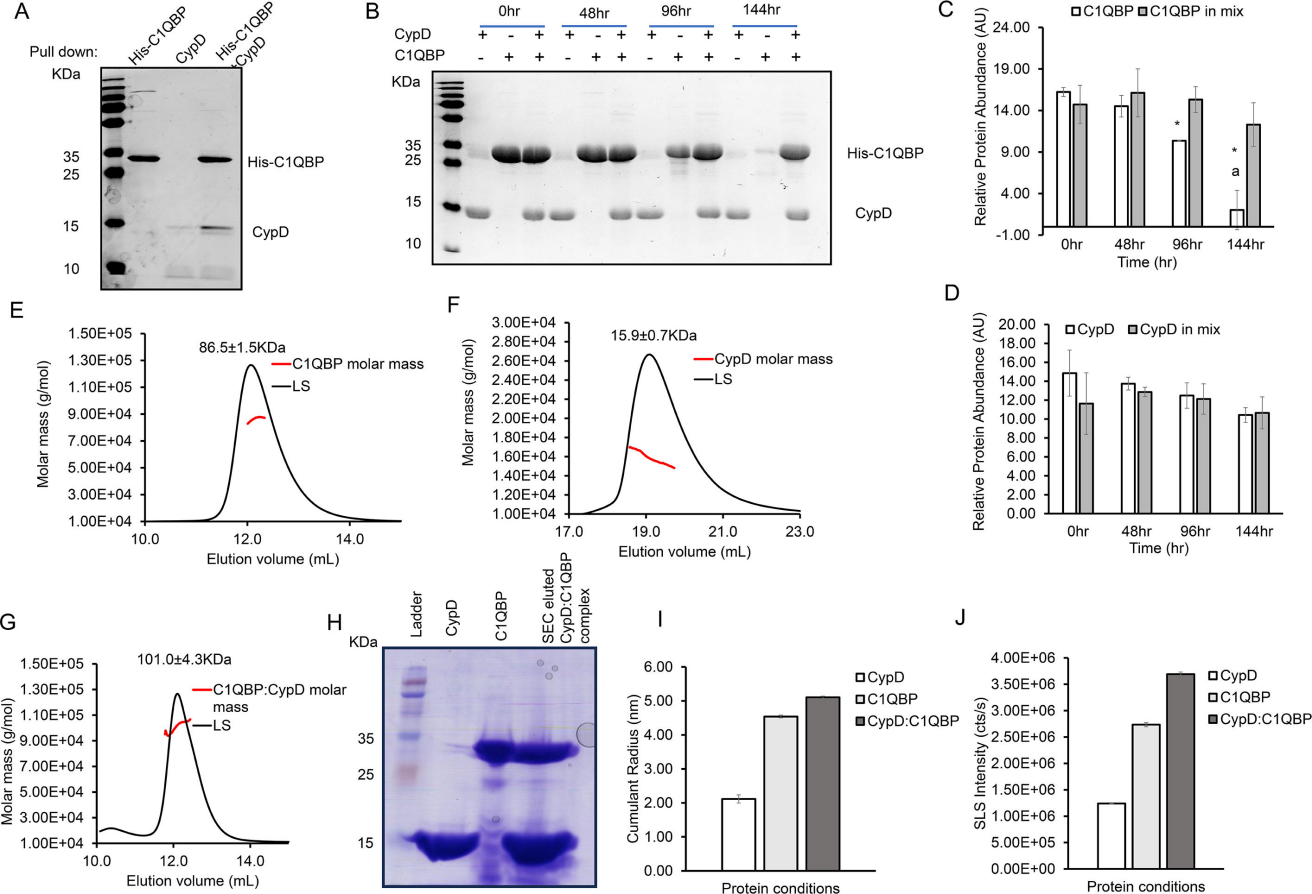
CypD stably interacts with C1qBP. (**A**) Coomassie-stained SDS–PAGE demonstrating the interaction between CypD and His-C1qBP in a pull-down assay. Lanes contain 10 µl of loaded eluate from each reaction condition. (**B**) Coomassie-stained SDS–PAGE of isolated and equimolar mixture of CypD and C1qBP over a 144-hour period at 37°C. (**C-D**) ImageJ densitometry measurements quantifying the relative percentage of protein abundance of both (**C**) C1qBP and (**D**) CypD in isolation and in combination (‘*’ denotes *P*<0.05 when compared with the control group, ‘a’ denotes *P*<0.05 when compared with C1qBP in mix). (**E-G**) Molecular mass analysis by SEC-MALS showing the fitted traces of (**E**) C1qBP at approximately 86 KDa, (**F**) CypD at approximately 16 KDa and (**G**) CypD:C1qBP complex at approximately 101 KDa. (**H**) Coomassie-stained SDS–PAGE of CypD, C1qBP and their SEC eluted complex (~10 µg loaded), confirming their presence and interaction. (**I-J**) DLS measurements of C1qBP and CypD in isolation and in complex, showing the (**I**) cumulant radius and (**J**) static light scattering (SLS). Bar charts display mean values (*n*=3) with corresponding standard error of the mean (SEM) error bars. Data were analysed using two-way Analysis of Variance (ANOVA) followed by Tukey’s post hoc test for multiple comparisons. An asterisk (*) indicates a statistically significant difference (*P*<0.05) compared with the 0-hour time point, while ‘a’ denotes *P*<0.05 compared with the mixed treatment within each group.

Protein pull-down experiments revealed that immobilised recombinant His-tagged C1qBP (referred to as C1qBP henceforth) captured recombinant CypD (Figure 2A). This result is in line with the findings of McGee and Baines [25] and other recent studies in which CypD was shown to co-precipitate with C1qBP.

The long-term stabilities of CypD and C1qBP, both individually and in equimolar mixture with each other, were monitored over the course of 144 hours at 37°C. Sample aliquots were collected for SDS–PAGE analysis every 48 hours. The results revealed that C1qBP steadily degraded over time (Figure 2B and C), while CypD remained significantly stable over the course of the 144-hour period (Figure 2B and D). Interestingly, over the same period, C1qBP when mixed with CypD was significantly more stable, suggesting that CypD stabilises C1qBP through the formation of a stable complex (Figure 2B and C) where, most likely, flexible regions of C1qBP prone to proteolytic cleavage become protected from this degradation.

The binding stoichiometry of CypD:C1qBP complex was determined using SEC-MALS. C1qBP and CypD were separately analysed first (Figure 2E and F). SEC-MALS revealed a trimeric recombinant C1qBP with molecular weight of ~86KDa (theoretical molecular weight (TMW) of trimeric Histag C1qBP is 80.8kD), while CypD is a monomer with SEC-MALs molecular weight of ~16 KDa (TMW is 17.8kD). Incubation of CypD:C1qBP in an excess molar ratio of CypD yielded a major eluted peak with a molecular mass of 101 kD (Figure 2G). This is consistent with one molecule of CypD binding to a trimeric C1qBP, resulting in a TMW of 98.6 kD. This observation aligns with previous reports of asymmetric binding between trimeric C1qBP and a monomeric partner [25–27]. CypD:C1qBP complex was collected from the SEC experiment and analysed by SDS–PAGE and DLS. The SDS–PAGE revealed the presence of both proteins in the sample (Figure 2H); it should be noted that Coomassie staining intensities are not directly comparable with concentrations of CypD and C1qBP present in the complex, as C1qBP exists as a trimer in its native state and has a higher molecular mass (~27 kDa per subunit) compared with monomeric CypD (~18 kDa). Consequently, 10 µg of C1qBP yields a lower apparent band intensity than the same mass of CypD, without contradicting the proposed stoichiometry of the complex. DLS of the SEC-isolated CypD:C1qBP complex showed an increase in cumulative radii (Figure 2I); likewise, SLS analysis revealed an increase in SLS by the complex (Figure 2J). These light scattering results further validated the formation of a stable complex.

**Figure 3:**
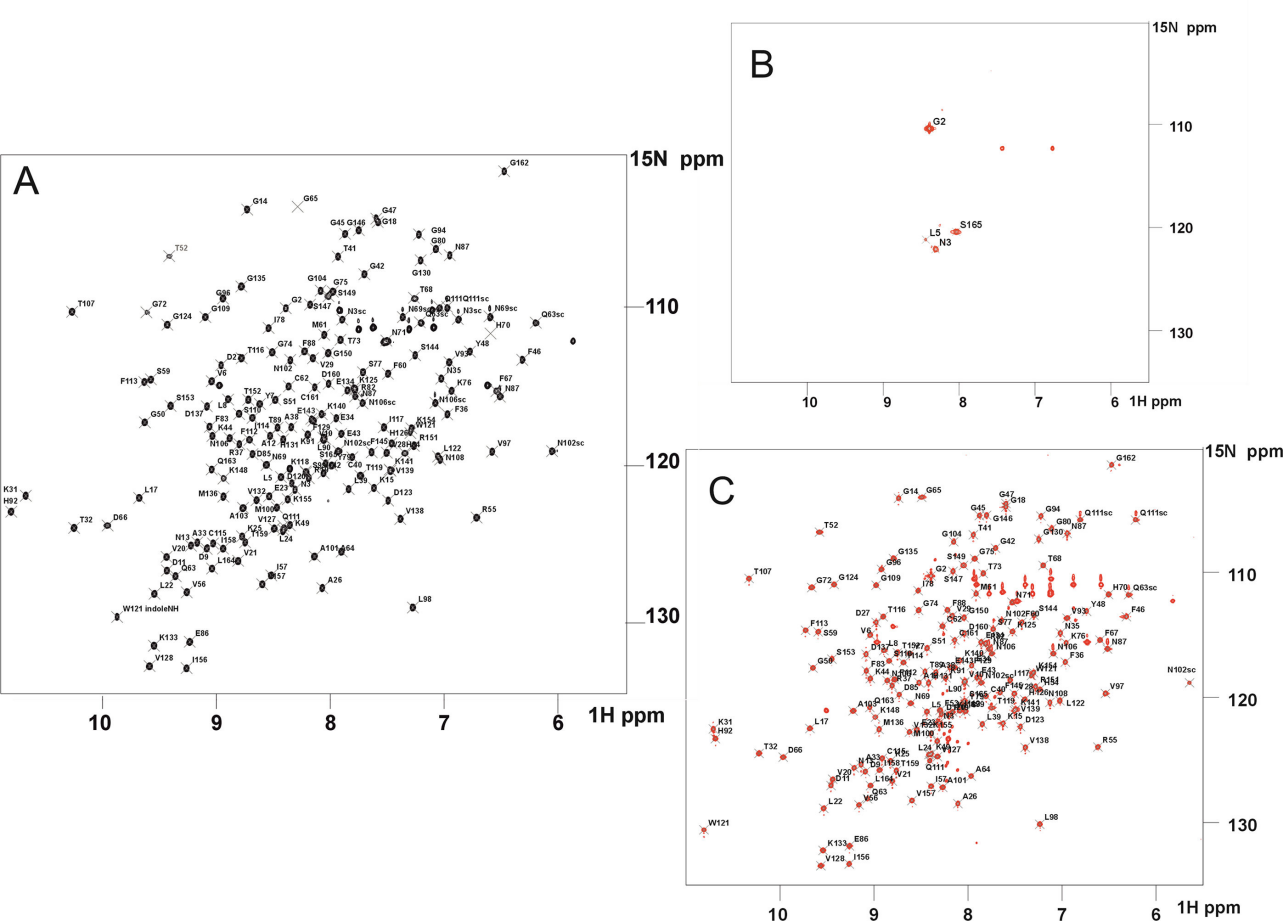
NMR Analysis of Interaction between CypD and C1qBP. Two-dimensional ^15^N-^1^H HSQC spectrum, 20 mM Na₂HPO₄, 20 mM NaCl, pH 6.5 298K of (**A**) ^15^N-uniformly labelled CypD alone, (**B**) ^15^N-uniformly labelled CypD in the presence of unlabelled C1qBP (CypD:trimeric C1qBP 4:1 molar ratio). (**C**) Spectrum of ^15^N-CypD:C1qBP complex following the addition of CsA (CypD:CsA 1:1 molar ratio). Assignments of these spectra were obtained in-house using standard triple resonance ^1^H, ^13^C, ^15^N experiments and compared with deposited assignments in the Biological Magnetic Resonance Databank.

**Figure 4:**
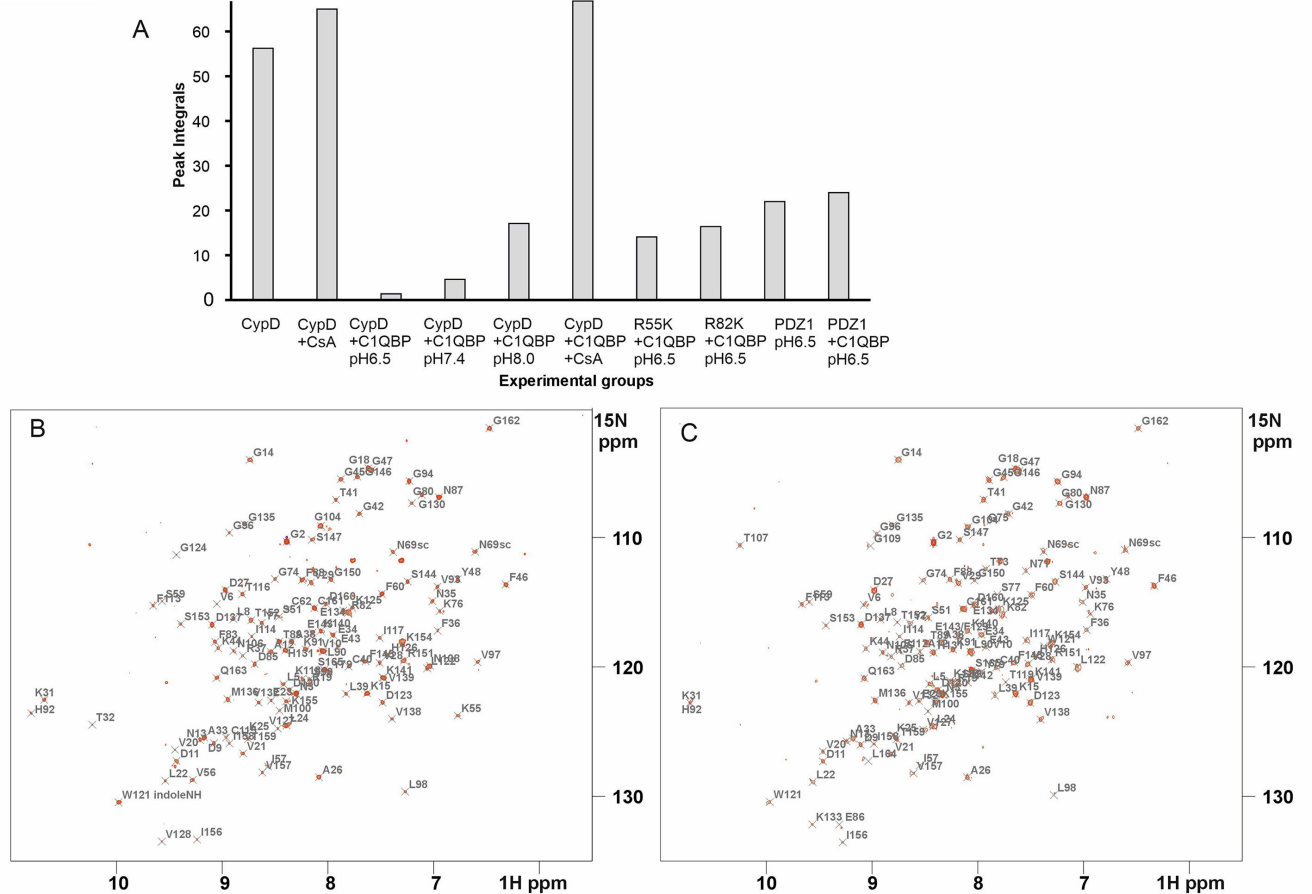
NMR Analysis of Interactions between CypD mutants and C1qBP. (**A**) Histogram showing total integrals of observable resonances of ^15^N-uniformly labelled CypD under the different sample conditions. Repeats were performed for the ^15^NCypD:C1qBP complex spectra, with the data showing similar intensities each time. (**B, C**) Two-dimensional ^15^N-^1^H HSQC spectrum, 20 mM Na₂HPO₄, 20 mM NaCl, pH 6.5 298K of (**B**) ^15^N-uniformly labelled CypD R55K, (**C**) ^15^N-uniformly labelled CypD R82K in the presence of unlabelled C1qBP (pH 6.5) (CypD:trimeric C1qBP 4:1 molar ratio). Assignments of these spectra were obtained in-house using standard triple resonance ^1^H, ^13^C, ^15^N experiments and compared with deposited assignments in the Biological Magnetic Resonance Databank.

Heteronuclear single-quantum coherence (HSQC) two-dimensional ^15^N-^1^H NMR spectroscopy was used to further characterise the interactions between C1qBP and CypD; ^15^N-^1^H HSQC NMR experiments were performed using recombinant uniformly ^15^N-labelled CypD and unlabelled C1qBP. In line with the SEC-MALS data, the NMR experiments confirmed stable interactions between CypD and C1qBP; the HSQC spectrum of ^15^N-labelled CypD at pH 6.5 was characterised by extensive loss of intensities for a majority of the CypD ^15^N-^1^H resonances in a 4:1 molar ratio of CypD:trimeric C1qBP mixture (Figures 3A, B and 4A). This severe global attenuation of signal intensities is likely due to the intermediate rate exchange regime on the NMR timescale between free and complexed CypD, coupled with the slow tumbling rates of the large CypD:C1qBP protein complex (Mr~101 KDa), which would lead to faster T_2_ relaxation and hence line-broadening of the complex resonances, relative to CypD alone (~16 KDa) [28,29].

These NMR results further revealed that CypD stably interacts with C1qBP. As a control experiment, 100 µM ^15^N-labelled PDZ domain of PSD95 (PDZ1) was mixed with 25 µM C1qBP; there was no difference in the HSQC spectra of ^15^N-labelled PDZ1 with or without C1qBP, indicating no complex formation between the two proteins ( Supplementary Figure S5, Figure 4A).

### CypD–C1qBP interaction is inhibited by inhibitor cyclosporin A (CsA)

Addition of CsA (in a 1:1 molar ratio to CypD) to the preformed CypD:C1qBP sample led to a total regain of the intensities of the CypD ^15^N-^1^H resonances of CypD (Figure 3C). The total integral of the CypD:CsA resonances is higher than that of CypD alone due to the stabilisation of some residues of CypD upon complexation with CsA (Figure 4A). These results indicate the complete dissociation of C1qBP from CypD by CsA and corroborate the findings of Baines and colleagues [9] showing that a CypD–CsA complex failed to bind to C1qBP.

The above results were cross-validated with active site mutants of CypD, R55K and R82K (R97 and R124 in the full-length CypD sequence, Uniprot P30405). R55 is a prime residue in the S1 ligand-binding pocket acting as a hydrogen-bond anchor to the carbonyl group of the peptidyl-prolyl bond to facilitate *cis-trans* isomerisation of that bond, whereas R82 modulates the size of the S2 pocket which in turn dictates substrate specificity among different cyclophilins [30]. Mutations at these sites have been reported to suppress the PPIase enzymatic activity of CypD [30–32]. Using the same experimental conditions as for the wildtype CypD, adding C1qBP to R55K CypD and to R82K CypD (1:4 ratio) resulted in a decrease in the attenuation of the CypD ^15^N-^1^H resonances; this is in line with the reduced affinities of these mutants for interacting ligands [33] (Figure 4B and C; see Supplementary Figures S6 and S7 for amide NH assignments of, respectively, CypDR55K and R82K). In summary, this data shows that CypD associates with C1qBP in its PPIase active site and CsA dissociates the CypD:C1qBP complex by competitively binding to CypD, displacing C1qBP from CypD.

### Electrostatic surface interaction potentiates CypD–C1qBP binding

**Figure 5:**
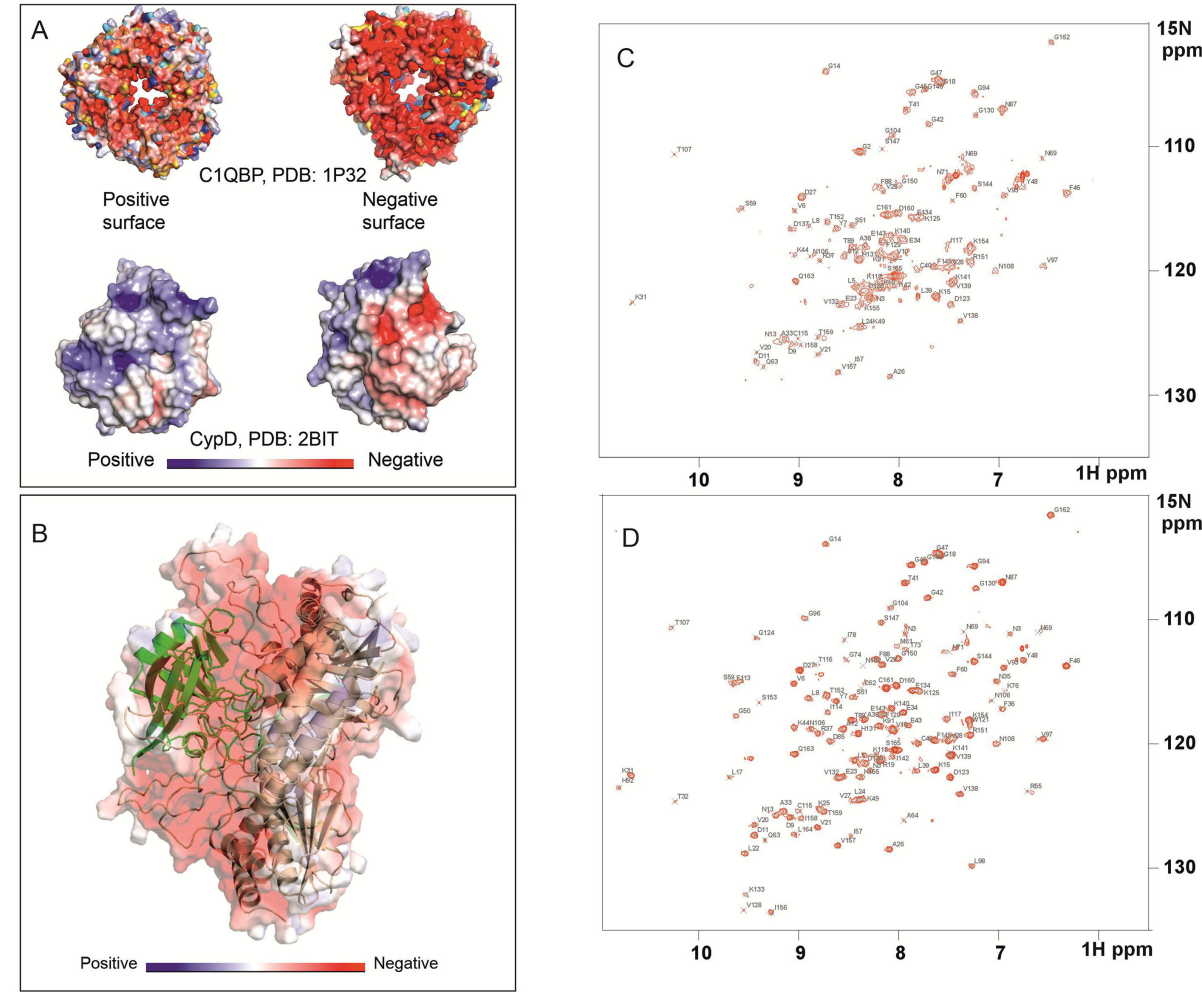
CypD–C1qBP binding is regulated by electrostatic interaction. (**A**) Adaptive Poisson–Boltzmann Solver (APBS) electrostatic surface analysis of trimeric C1qBP (PDB: 1P32) and CypD (PDB: 2BIT). Electrostatic potential mapping reveals distinct positive (blue) and negative (red) surface regions for both proteins. The colour bar is shown at the bottom of the panel. (**B**) AlphaFold-predicted interaction between CypD (green) and C1qBP (wheat), with the electrostatic surface overlaid over the cartoon representation. The colour bar is shown at the bottom of the panel. (**C, D**) Two-dimensional ^15^N-^1^H HSQC spectra, 20 mM Na₂HPO₄, 20 mM NaCl, 298K of uniformly ^15^N-labelled CypD in the presence of unlabelled C1qBP at pH 7.4 (**C**) and pH 8.0 (**D**).

The AlphaFold model [34–36] reveals interactions between CypD and trimeric C1qBP in which the positively charged surface of CypD is associated with the negatively charged surface of C1qBP (Figure 5A and B) [37] which includes intrinsically disordered regions, supporting the previously mentioned protection of labile C1qBP regions from proteolytic cleavage by CypD binding. To demonstrate the electrostatic and polar nature of the CypD–C1qBP interactions, the influence of pH on the stability of the complex was investigated. 2D ^15^N-^1^H HSQC spectra of ^15^N-labelled CypD in a 4:1 molar ratio of unlabelled C1qBP were acquired at pH values 6.5, 7.4 and 8.0. The number of observable ^15^N-^1^H resonances increased progressively with pH (Figures 3B–5C and 5D), implying that the strength of interactions decreased with increasing pH. The theoretical pI values of the C1qBP and CypD constructs used in this study were calculated as ~ 4.5 and 9.0, respectively, calculated using ProtParam [38,39]. An increase in pH decreases the electropositivity of CypD, leading to an expected reduction in C1qBP affinity.

**Figure 6:**
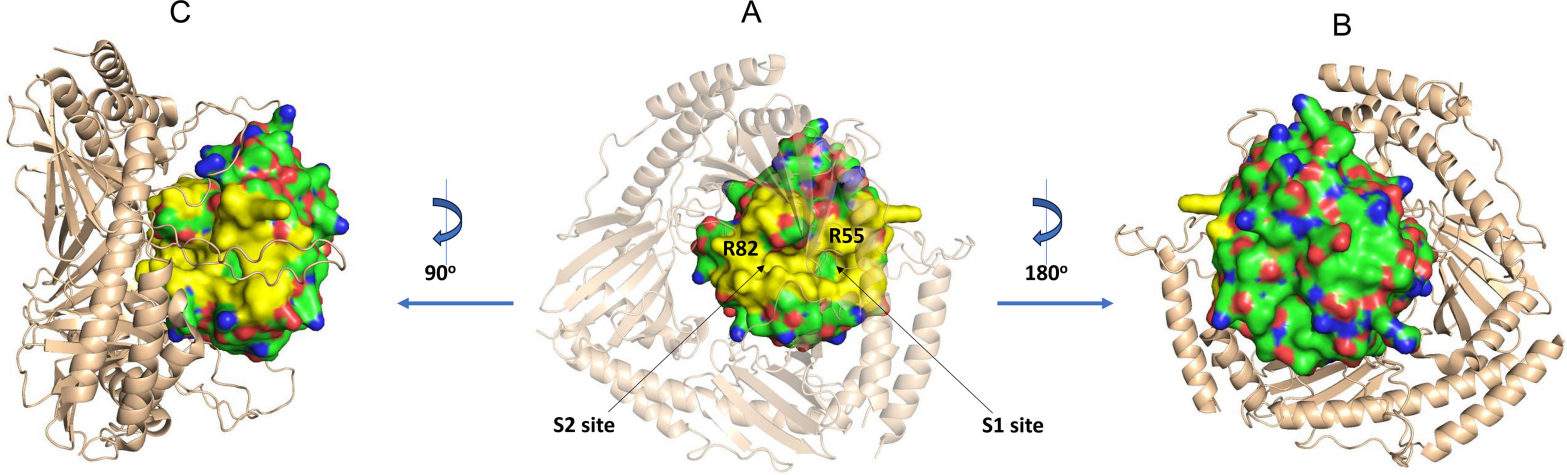
NMR chemical shift mapping of C1qBP binding surface of CypD. (**A**). Molecular surface of AlphaFold model of CypD in complex with C1qBP. Residues that show significant attenuation of NMR ^15^N-^1^H HSQC signal intensities at pH 8.0 are coloured in yellow, with a transparent cartoon representation of C1qBP (coloured in wheat) in the foreground. (**B**) 180° rotation of **A** to show the reverse surface of CypD, with C1qBP in the background, highlighting little chemical shift perturbations in the complex ^15^N-^1^H HSQC spectrum. (**C**) 90° rotation of **A** to show the interactions involving C1qBP acidic loop N61–F95 from one of the subunits of the trimer. The structures were created using the program PyMOL (Schrödinger, LLC).

Analyses of the resonances that are significantly line-broadened at pH 8 allowed the identification of the CypD binding surface with C1qBP. These residues are R55, V56, I57, S59, F60, M61, C62, Q63, A64, D66, F67, T68, N69, H70, N71, G75, K76, S77, I78, G80, S81, R82, F83, M100, A101, N102, A103, N108, G109, S110, Q111, W121 (indole NH), L122, H126, K148 and S149. When mapped onto the structure of CypD, these residues are confined to one side of CypD on the surface that forms the interacting face of CypD with C1qBP in the AlphaFold model (Figures 6A and B). Many of these residues surround the S1 and S2 binding pockets, which include residues R55 and R82 and constitute the positive surface of CypD (Figure 6A). Resonances of some residues just beyond the S1 site are also significantly attenuated; interestingly, the AlphaFold model shows a long, glutamate-rich loop of one subunit N61–F95 of C1qBP positioned adjacent to this region of CypD (Figure 6C).

These results clearly indicate that electrostatic/polar interactions potentiate CypD–C1qBP interaction. Since mitochondria undergo pH oscillations between pH~7.2–8.2 during various metabolic and physiological states [40–44], the results here suggest that mitochondrial matrix pH status may play a role in the stability of CypD–C1qBP interaction *in vivo*.

### C1qBP sequesters calcium

**Figure 7:**
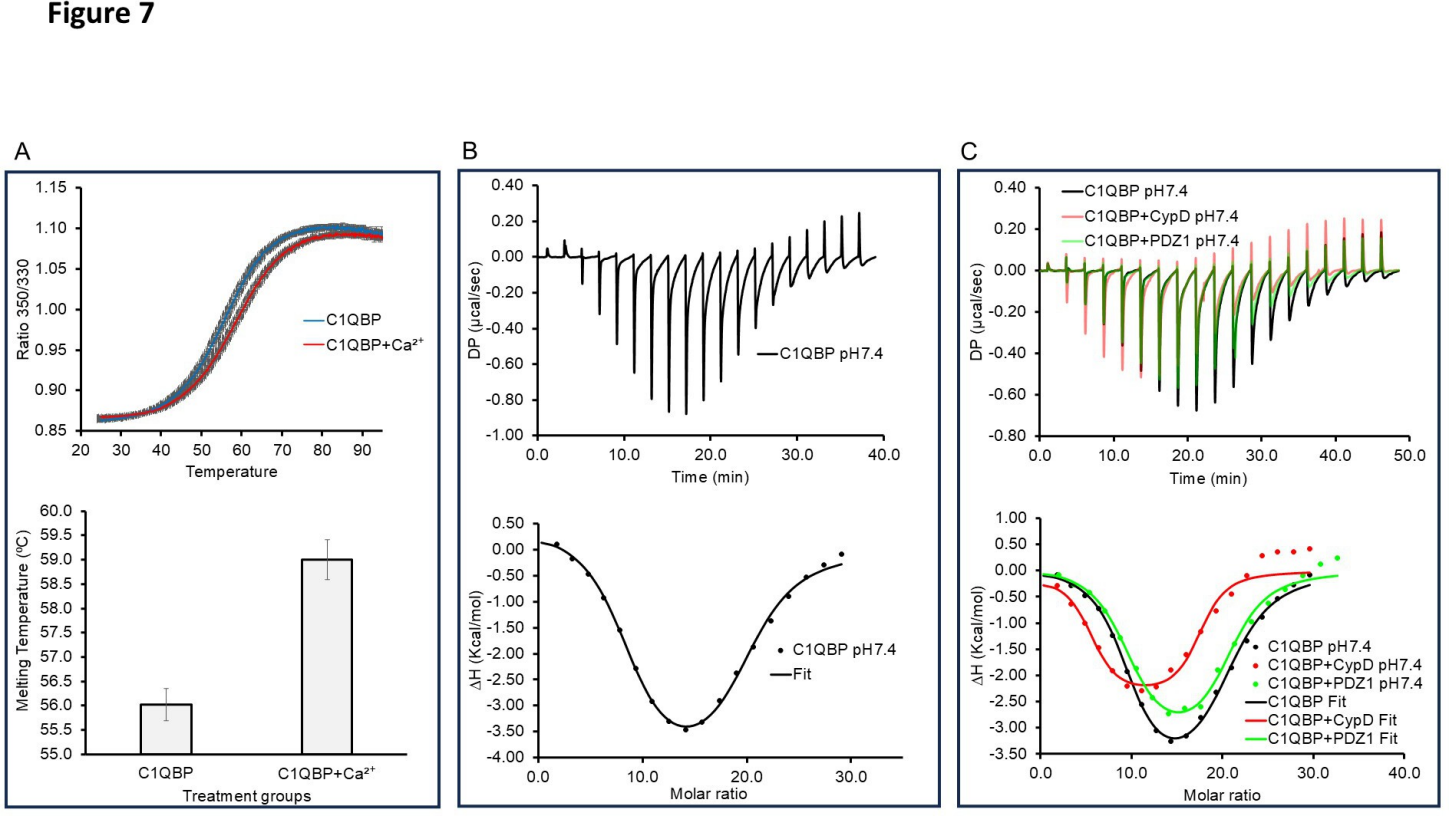
C1qBP binds Ca2^2+^ and CypD suppresses its capacity. (**A**) NanoDifferential Scanning Fluorimetry (NanoDSF) thermal unfolding profiles of C1qBP (0.8 mg/ml; 30 µM) without and with Ca^2+^(2.5 mM) showing a 3°C increase in melting temperature (Tm), indicating Ca^2+^-dependent stabilization (*P*<0.001). The accompanying bar chart quantifies this shift (bar charts represent mean values (*n*=4) with corresponding SEM error bars. Welch’s two-sample t-test was used to compare group means). (**B**) Isothermal titration calorimetry (ITC) of C1qBP binding to Ca^2+^ at pH 7.4, revealing biphasic binding kinetics with two distinct stoichiometries. (**C**) ITC overlay comparing Ca^2+^ binding to C1qBP alone, in the presence of CypD and with a negative control (PDZ1), demonstrating reduction in the binding capacity of C1qBP for Ca^2+^ in the presence of CypD. Thermodynamics values are representative of mean ± SEM, (*n* ≥ 2).

C1qBP, with its distinct negatively charged surface, had been hypothesised to bind to calcium and other metal ions [20,21,45,46], although direct biochemical evidence to substantiate this was lacking. Here, differential scanning fluorimetry (DSF) data showed an elevation of C1qBP melting temperature by ~3°C following the addition of calcium (2.5 mM) to protein (~30 µM), indicating that calcium binds to recombinant C1qBP (Figure 7A).

The binding kinetics of C1qBP to Ca^2+^ were investigated using isothermal titration calorimetry (ITC) at pH 7.4. The ITC isotherm showed complex kinetics, with at least two phases of binding (Figure 7B). Using two sets of site analyses for the binding, at pH 7.4, the thermodynamics values obtained for the two sites corresponded to the two discernible phases (Table 1). Both binding phases appear to consist of endothermic and exothermic processes, with their proportions varying over the course of the Ca^2+^ titration. The first, high-affinity binding phase shows decreasing endothermic and increasing exothermic processes, with entropic changes being the main driving force, most likely due to conformational changes of C1qBP. Binding to the second, low-affinity sites is enthalpically driven, albeit less favourably than the entropic binding. The high N values of 9 and 12 for, respectively, the first and second phases indicate that C1qBP is likely to sequester Ca^2+^, binding Ca^2+^ using multiple binding sites. This is due to the extensive negatively charged surface on C1qBP and its lack of consensus Ca^2+^ binding motifs, confirmed by the ion prediction programme IonCom [47] to confer specific Ca^2+^ binding. The increasing endothermic spikes as the titration approached saturation indicated further C1qBP conformational changes in the presence of very high Ca^2+^ concentrations.

**Table 1:** Thermodynamics parameters^($)^ describing the interaction between C1qBP and calcium in the absence and presence of CypD

Thermodynamics parameters	C1qBP	C1qBP:CypD
K_d1_ (µM)	0.48 + 0.01	0.14 + 0.01
ΔH_1_ (kcal/mol)	0.25 + 0.22	0.09 + 0.002
-TΔS_1_ (kcal/mol)	-8.87 ± 0.21	-9.29 + 0.03
N_1_	9	5
K_d2_ (µM)	10.90 + 1.33	3.54 + 0.15
ΔH_2_ (kcal/mol)	-4.14 + 0.19	-2.22 + 0.28
-TΔS_2_ (kcal/mol)	-2.63 ± 0.11	-5.16 ± 0.23
N_2_	12	12

^($)^ Thermodynamic values are presented as mean ± SEM (Standard Error of the Mean), calculated from replicate measurements of n ≥ 2

**Figure 8:**
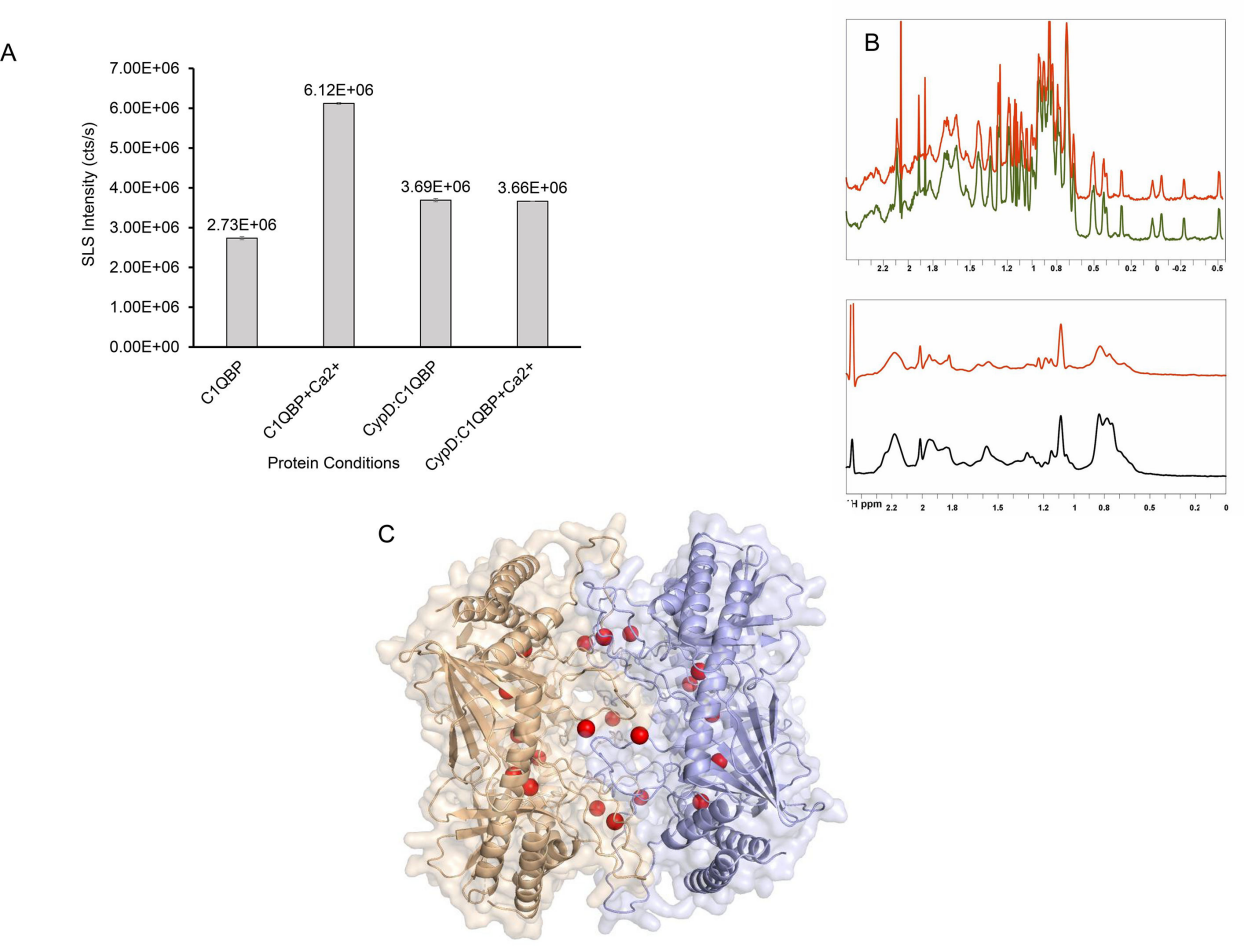
(A) Static light scattering (SLS) analysis of C1qBP and the CypD:C1qBP complex in the absence and presence of calcium. Bar graphs represent mean values (*n*=3) with error bars indicating the standard error of the mean (SEM). (B) 1D ^1^H NMR spectra of CypD (upper) in the absence (green) and presence (red) of 15 mM Ca^2+^, and of C1qBP (with substoichiometric amounts of CypD) (lower) in the absence (black) and presence (red) of 15 mM Ca^2+^. The intensities of the peaks are normalised to proton resonances from buffer additives. (C) AlphaFold-predicted model of hexameric C1qBP showing sequestration of calcium ions within the inter-trimeric space. The two trimeric C1qBP units are depicted in wheat and light blue, respectively, while Ca^2+^ is depicted in red.

Light scattering experiments provided evidence for this possible change in C1qBP conformation: Ca^2+^:C1qBP exhibited an increase in cumulant dynamic scattering radius and an approximately two-fold rise in SLS intensity (Figure 8A). Ca^2+^:C1qBP appears to adopt a higher multimeric state, likely corresponding to a dimer of C1qBP trimers when compared with the non-Ca^2+^ bound protein. The 1D NMR spectrum of C1qBP also showed significant line-broadening when Ca^2+^ was added to the sample C1qBP (Figure 8B). Such Ca^2+^-induced hexamerisation may indicate that C1qBP sequesters Ca^2+^ by locking it within the complex, thereby functioning as a Ca^2+^ store and enhancing its overall binding capacity. AlphaFold3 modelling concurs with this hypothesis, predicting multiple Ca^2+^ ions binding at the interphase of a C1qBP trimer–trimer complex, generating an enclosed architecture (Figure 8C) [48], further supporting the proposed role of C1qBP in Ca^2+^ sequestration.

The complicated binding thermodynamics observed here are characteristics of co-operative ligand binding, as ligand association with these proteins often entails heat changes resulting from, simultaneously or sequentially, electrostatic interactions, local and/or gross conformational changes [49,50].

### CypD prevents efficient calcium sequestration by C1qBP

C1qBP complexed to CypD bound Ca^2+^ (at pH 7.4) with a similar thermodynamics profile as uncomplexed C1qBP (Figure 7C). Two sets of site analyses of the thermodynamics values again showed two discernible binding phases (Table 1). Notably, CypD reduced the stoichiometry of the Ca^2+^ high-affinity binding sites in C1qBP by approximately 50% (N₁=5 vs. 9 for C1qBP:Ca^2+^).

Additionally, SLS analysis showed similar scattered light intensities for the Ca^2+^:CypD:C1qBP and C1qBP:CypD complexes, indicating that Ca^2+^ had no effect on the molecular weight of the complex as opposed to the two-fold increase in light intensity when it bound to C1qBP alone (Figure 8A).

Collectively, both the ITC (drop in overall N value of the complex) and light scattering data indicated that CypD inhibited Ca^2+^-induced conformational changes of C1qBP and reduced the number of available Ca^2+^ binding sites, effectively diminishing the Ca^2+^-sequestration capacity of C1qBP. Additionally, the leftward shift of the trough in the ITC binding isotherm indicated a lower molar ratio of Ca^2+^ to the protein complex, further supporting this impaired sequestration of Ca^2+^ (Figure 7C). PDZ1 was used as a negative control; addition of PDZ1 to C1qBP did not significantly alter the N value of the high-affinity set of sites (N1=~ 9) (the slight reduction in ΔH may be due to a difference in protein concentrations). It is worth noting that neither CypD nor PSD95 PDZ1 binds Ca^2+^ as monitored by ITC (Supplementary Figures S8 and S9) and NMR (for CypD; Figure 8B).

In summary, these results suggest that C1qBP undergoes dynamic conformational changes upon Ca^2+^ binding, promoting multimerisation and creating additional binding sites and storage for Ca^2+^. CypD appeared to inhibit this multimerisation, thereby reducing the overall Ca^2+^ binding capacity of C1qBP.

## Discussion

The direct interaction between CypD and C1qBP is described and characterised here. C1qBP binds to the putative ligand-binding sites of CypD, since the binding is inhibited by the classic cyclophilin inhibitor, CsA. Furthermore, CypD–C1qBP binding affinities were decreased in the CypD R55K and R82K mutants; both R55 and R82 are crucial ligand-binding residues, forming hydrogen bonds with ligands and, in the case of R82, modulating the size of the ligand-binding pocket.

From their three-dimensional structures, CypD and C1qBP have, respectively, an extensive positively and negatively charged surface. AlphaFold indeed predicts that the positively charged surface of CypD binds to the negatively charged surface of C1qBP which is formed by its long stretches of intrinsically disordered regions, especially the acidic region N63–F95 (N134–F168 based on the UniProt accession number, Q07021). The NMR experiments validated this model; binding between CypD and C1qBP was shown to progressively weaken with increasing pH values, and analyses of the selective line-broadened resonances allowed the mapping of the C1qBP-binding face of CypD to the AlphaFold-predicted face.

Based on its distinct dipole characteristics, C1qBP had been hypothesised to bind to Ca^2+^; this binding is characterised here in detail for the first time. In the absence of consensus Ca^2+^-binding sequences within C1qBP, it is likely that its large negatively charged protein surface enables it to chelate many ions such as Ca^2+^, as predicted by the IonCom programme [47]. The ITC data obtained here show that C1qBP binds calcium with high capacity and low affinity, implying that C1qBP most likely acts as a calcium sequester/storage. Exposure to Ca^2+^ appears to lead to conformational changes which predispose C1qBP to make available more Ca^2+^ binding sites prior to saturation.

The hypothesis that C1qBP could be a Ca^2+^ chelator/store may explain the earlier observations made by McGee and Baines in which C1qBP was shown to inhibit mPTP activation and that C1qBP overexpression enhanced mitochondria capacity to retain calcium after ROS treatment of MEF cells [9,23], as well as why certain cancer cells overexpress C1qBP as an adaptive response [51–55]; these observations concur with C1qBP acting as a Ca^2+^ sequester, with cells avoiding Ca^2+^-induced death amid their elevated calcium-dependent pathways.

In this study, the presence of CypD was shown to lower the sites available on C1qBP for Ca^2+^ binding, effectively diminishing its Ca^2+^ sequestration capabilities. This effect of CypD on Ca^2+^–C1qBP interactions makes sense, as CypD is experimentally shown here to interact with the same negative face of C1qBP as the Ca^2+^. CypD reduces the stoichiometry of high-affinity sites by approximately half, without affecting low-affinity sites, suggesting a significant functional modulation. By limiting high-affinity Ca^2+^ binding, CypD thus lowers the capacity of C1qBP to act as a Ca^2+^ sink under conditions of overload. The observations presented here potentially provide an explanation for the role of CypD in the mPTP formation, a phenomenon with which the biological significance of CypD is most clearly associated. It is known that following genetic ablation of the *PPIF* gene (which codes for CypD), higher Ca^2+^ levels were required to disrupt mitochondrial membrane potential through the formation of the mPTP; the reduction of C1qBP Ca^2+^ binding capabilities shown here may provide an explanation for this observation [56].

**Figure 9:**
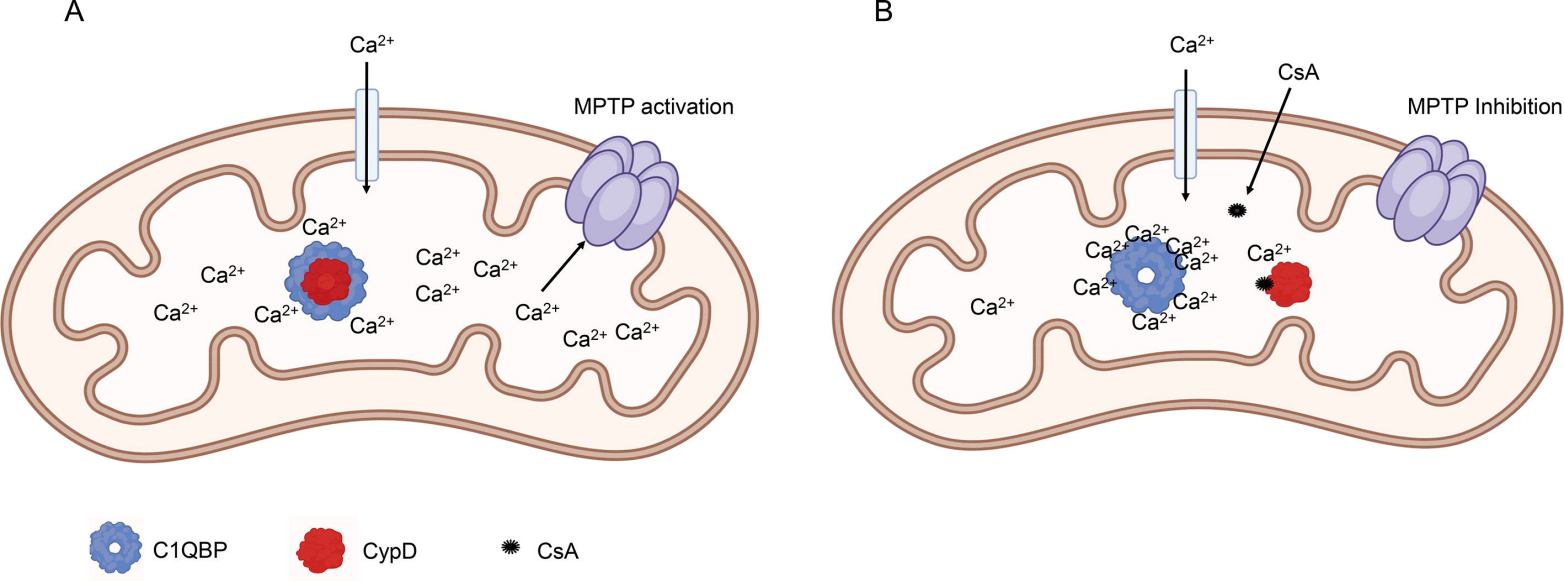
Proposed model of mPTP regulation by CypD and C1qBP interaction. (**A**) In the presence of CypD, the ability of C1qBP to sequester Ca^2+^ is impaired, leading to excessive mitochondrial matrix calcium accumulation, which subsequently triggers mPTP activation. (**B**) CsA binding to CypD dissociates the CypD:C1qBP complex, restoring the capacity of C1qBP to sequester and store Ca^2+^, thereby lowering matrix Ca^2+^ levels and preventing mPTP activation, which would otherwise require excessive Ca^2+^ following C1qBP saturation for reactivation. This model highlights the dynamic regulation of mPTP by CypD–C1qBP interactions and Ca^2+^ availability. The figure was created in BioRender.com.

We propose a model which shows C1qBP functioning as a Ca^2+^ sequester protein and a potential mechanism for the role CypD plays as a regulator of the mPTP (Figure 9). In this model, CypD impairs C1qBP Ca^2+^ binding, allowing free Ca^2+^ to accumulate, ultimately triggering mPTP activation (Figure 9A) [7,57,58]. In the presence of CsA, CypD dissociates from C1qBP, enabling C1qBP to sequester Ca^2+^ and no mPTP activation; however, under excessive Ca^2+^ accumulation, when the capacity of C1qBP Ca^2+^ sequestration is surpassed, the free Ca^2+^ will eventually cause mPTP formation (Figure 9B). This model aligns with the known key characteristics of mPTP regulation.

In summary, this study shows that C1qBP is an efficient Ca^2+^ sequester and undergoes conformational changes to accommodate and stabilise high numbers of Ca^2+^ within a complex, effectively forming a calcium storage unit. On the other hand, the presence of CypD diminishes C1qBP capacity for effective Ca^2+^-binding via conformational changes and competition for the same negative surface of C1qBP. This *in vitro* study is one of the few demonstrations of the direct interactions between two mitochondrial matrix proteins, one of which sequesters Ca^2+^. There are parallels between the characteristics of CypD–C1qBP interactions uncovered here and the regulation of Ca^2+^ induced opening of mPTP by CypD; this should provide fresh avenues for further investigations into the identity and regulation of the mPTP.

## Materials and methods

### Protein expression and purification

Mature human mitochondrial CypD corresponding to residues 44–207 of the fully translated gene sequence (UniProt P30405) was cloned into the pETM11 (EMBL) expression vector, which includes a hexahistidine Ni^2+^ affinity tag (His-tag), as previously reported [32]. CypD R55K and R82K point mutations were introduced into the *PPIF* gene for CypD as described previously [32] using the Agilent QuikChange II XL site-directed mutagenesis kit (#200521) and the described protocol, with the plasmid pETM11 CypD as a template. Human C1qBP corresponding to residues 75–282 of the fully translated gene sequence (UniProt Q07021) was cloned into the pETM11 (EMBL) expression vector. The following primers were used for cloning C1qBP:

C1qBP Forward primer: TCAGGGCGCCATGCACACCGACGGAGACAAAGC

C1qBP Reverse primer: CGGATCCCTATCACTGGCTCTTGACAAAACTCTTGAG

pETM11 backbone reverse primer: CGGTGTGCATGGCGCCCTGAAAATAAAGATTC

pETM11 backbone forward primer: GAGCCAGTGATAGGGATCCGAATTCGAGCTCCGT

Details of the insert sequence for Gibson assembly cloning for mature C1qBP, and for CypD R55K and R82K mutations are provided in the Supplementary 1–3.

CypD and PDZ1 production has been previously reported [32,59]. Recombinant CypD and its R55K and R82K mutants lacked residues 1–13 at the N-terminus of mature CypD, as this has been shown to retain full functionality [8,30,60]. CypD, C1qBP and PDZ1 were heterologously expressed in *Escherichia coli* BL21 (DE3) star strains. All expression media described here contain kanamycin. The BL21 (DE3) star cells were transformed with the respective expression plasmids, plated on Luria-Bertani (LB) agar and incubated overnight at 37°C. A single colony was used to inoculate 1 ml of LB broth, which was grown for 5 hours at 37°C with shaking. Subsequently, 100 µl of this culture was transferred into 20 ml of M9 media to make a starter culture, incubated overnight at 37°C. The starter culture (20 ml) was then used to inoculate 1 l of M9 media, which was grown at 37°C and 210 rpm until an OD600 reached 0.7. Protein expression was induced with 0.8 mM isopropyl β-D-thiogalactopyranoside, and the cultures were incubated overnight at 18°C with shaking at 180 rpm. Cells were harvested by centrifugation at 5000 rpm at 4°C. For preparing ^15^N isotopically labelled proteins for 2D NMR experiments, the protein expression minimal media was made using ^15^NH_4_Cl.

The cell pellet was resuspended to 5% w/v in buffer A (50 mM Tris pH 7.4, 300 mM NaCl, 5% glycerol) supplemented with 15 μg/ml DNase1, incubated on ice for 20 minutes, and lysed using a cell disruptor (Constant Systems, U.K) at 19 kilo-pounds per square inch (KPSI) and 4°C. The lysate was centrifuged at 18,000 rpm, 4°C, and the supernatant was passed through a pre-equilibrated 5 ml FF His-trap column (Cytiva, U.S.A.), washed with 5 column volumes of 5% buffer B (buffer A + 500 mM imidazole) and the protein was eluted using a gradient of buffer B. Protein samples were further purified via SEC using a Superdex 200 16/600 column (GE Healthcare) in a buffer containing 50 mM Na_2_HPO_4_ pH 7.4, 150 mM NaCl, and 5% glycerol. The purified proteins, with a yield of between 10 ~ 20 mg/l for C1qBP and CypD wildtype and mutants, were aliquoted at ~2 mg/ml and stored at -80°C. For experiments, protein samples were buffer exchanged into either buffer C (20 mM Na_2_HPO_4_ pH 6.5 or 7.4, 20 mM NaCl – for experiments not involving Ca^2+^), or buffer D (20 mM HEPES pH 7.4 – for experiments involving Ca^2+^). Expression of the newly created construct for C1qBP was confirmed by Western blotting analyses (Supplementary Figure S4). Detection was performed using a rabbit anti-C1qBP antibody (Proteintech, U.S.A., Cat. No. 24474–1-AP).

### Pull-down assay

Nickel-Nitrilotriacetic acid (Ni-NTA) agarose beads (30 µl slurry) were equilibrated by washing twice with 500 µl binding buffer (20 mM Na_2_HPO_4_, 20 mM NaCl, pH 7.4). Washes were performed by centrifugation at 5,000 rpm for 1 minute at 4°C and removal of the supernatant. Binding reactions (total volume 100 µl) contained binding buffer and the following protein inputs: 50 µg His-C1qBP, 150 µg CypD, or both proteins combined (50 µg His-C1qBP + 150 µg CypD). Reaction mixtures were incubated at room temperature for 20 minutes and then applied to the equilibrated Ni-NTA beads. Samples and beads were incubated at room temperature for a further 20 minutes with occasional mixing by pipetting. Beads were pelleted by centrifugation at 5,000 rpm for 1 minute at 4°C and the supernatant was discarded. Beads were washed twice with 500 µl binding buffer containing 25 mM imidazole (centrifugation at 5,000 rpm for 1 minute at 4°C between washes). Bound material was eluted in 30 µl binding buffer containing 500 mM imidazole. Each sample eluate (15 µl of supernatant mixed with 5 µl 4× SDS–PAGE sample buffer) was made and 10 µl was loaded per lane for SDS–PAGE analysis.

### Thermostability assay

Thermostability of CypD and C1qBP was assessed by monitoring protein integrity over extended incubation. Recombinant CypD and C1qBP (100 µM each) were prepared individually and as equimolar mixtures in binding buffer (20 mM Na_2_HPO_4_, 20 mM NaCl, pH 7.4) in a total reaction volume of 500 µl. Samples were incubated at 37°C in a static incubator for up to 144 hours. Aliquots (10 µl) were collected at 48 hours intervals, diluted with 5 µl deionised water and mixed with 5 µl 4× SDS–PAGE sample buffer. Aliquots were immediately frozen and stored at −20°C until analysis. Samples were subsequently thawed, denatured and analysed by SDS–PAGE. Band intensities were quantified using ImageJ software for densitometry analysis.

### Size exclusion chromatography-multi-angle light scattering (SEC-MALS)

Recombinant CypD and C1qBP were concentrated to 2–4 mg/ml and filtered through a 0.22 µm syringe filter in Buffer C, pH 6.5. For SEC-MALS analysis, 200 µl of either the individual protein solutions or a CypD:C1qBP mixture at a 2:1 molar ratio was injected into a Superdex 200 10/300 GL column pre-equilibrated with 20 mM Na₂HPO₄, 20 mM NaCl, pH 6.5. The column was connected in line with MALS and differential refractive index (dRI) detectors. The flow rate was maintained at 0.75 ml/min throughout the analysis. Prior to sample analysis, the MALS and dRI detectors were calibrated using bovine serum albumin as a standard. Data were collected simultaneously from the MALS, dRI and UV detectors. The collected data were analysed using the ASTRA software (Wyatt Technologies, U.S.A.) to determine the molar mass and hydrodynamic radius of the eluted protein samples.

### NMR spectroscopy

Uniformly ^15^N-labelled recombinant CypD was diluted to a final concentration of 100 µM in 600 µl of buffer C (20 mM Na₂HPO₄, pH 6.5, 7.4 or 8.0, 20 mM NaCl) with 10% v/v D2O. Binding studies were conducted by mixing the ^15^N-labelled CypD with CsA at a 1:1 molar ratio or with unlabelled recombinant C1qBP at a 4:1 molar ratio (CypD:trimeric C1qBP) and pre-equilibrated at room temperature before data collection. All ^15^N-HSQC NMR experiments were performed at 298K using a Bruker Avance III 700 MHz spectrometer equipped with a TCI cryoprobe [1H, ^15^N, ^13^C] and deuterium frequency lock. Water suppression was achieved through excitation sculpting. Data analysis was carried out using CcpNMR Analysis version 3.1.1.

### Isothermal titration calorimetry (ITC)

The experiments were conducted using a Malvern Microcal PEAQ Automated system, controlled by its proprietary software, MicroCal PEAQ_ITC Automated Control Software. The instrument was configured for 19 injections at 25°C, with the reference power set to 6 µcal/s, an initial delay of 60 seconds and a stir speed of 750 rpm. The cell contained 20 µM protein – C1qBP, CypD, PDZ1, CypD:C1qBP equimolar mix, or PDZ1:C1qBP equimolar mix in 20 mM HEPES pH 7.4 (while the syringe held 2.5 mM CaCl_2_). A Ca^2+^-to-buffer titration served as the internal control. Both the protein and ligand were diluted in buffer D (20 mM HEPES, pH 7.4). The initial injection volume was 0.4 µl, followed by subsequent injections of 2 µl. Data evaluation was performed using MicroCal PEAQ-ITC Analysis Software.

### Dynamic light scattering and differential scanning fluorimetry

Recombinant proteins C1qBP, CypD and SEC-eluted CypD:C1qBP complex were prepared at a final concentration of 0.8 mg/ml. These samples were prepared in buffer D at pH 7.4, both in the presence and absence of 2.5 mM CaCl_2_. Each protein sample was carefully loaded into nanoDSF capillaries and subsequently inserted into a Prometheus Panta nanoDSF instrument (NanoTemper Technologies, Germany). For the DLS analysis, the temperature was maintained at 37°C to assess the size distribution of the protein samples. For DSF analysis, a thermal gradient from 25°C to 95°C was applied to evaluate the thermal stability of the proteins. The measurement protocol was initiated to simultaneously record intrinsic fluorescence signals and DLS data of the samples. The DLS data were analysed to determine the size distribution of the proteins and protein complexes, while the DSF data were evaluated to assess the thermal stability profiles.

### Structural prediction of C1qBP interaction with CypD and calcium ions

To investigate the interaction between C1qBP and either CypD or Ca^2+^, a structural model of the protein complex was generated using AlphaFold3 [34–36]. Notably, both AlphaFold 2 and 3 predictions of C1qBP showed no difference in the predicted structure (Supplementary Figure S10). Protein sequences for CypD and C1qBP were retrieved from their respective entries in UniProt (CypD, P30405, residues 43–207; C1qBP, Q07021, residues 74–282) and used as input for the ColabFold and AlphaFold3 server using default settings. For modelling Ca²^+^ binding, calcium ions were selected under the ion tab and docked with the hexameric form of C1qBP. Among the predicted models, the model with high predicted local distance difference test scores and inter-residue confidence metrics was selected. The resulting structural models of the CypD:C1qBP complex and C1qBP:Ca^2+^ have been deposited in the Model Archive database (modelarchive.org) [61] under the accession codes ma-0taq4 and ma-4mxcf, respectively, and is publicly accessible via the weblinks https://www.modelarchive.org/doi/10.5452/ma-0taq4 (password: fy0r32B9uz) [37] and https://www.modelarchive.org/doi/10.5452/ma-4mxcf (password: 0FE2npnZET) [48]

### Electrostatic surface potential analysis

Electrostatic surface potentials were computed to identify regions of complementary charge distribution that may facilitate the interaction between CypD and C1qBP. Individual crystal structures (CypD:2BIT; C1qBP: 1P32) and the AlphaFold-predicted CypD:C1qBP complex were analysed. All structures were prepared for electrostatic analysis using the PDB2PQR server with AMBER force field parameters to assign atomic charges and radii. Electrostatic potentials were then calculated using the Adaptive Poisson–Boltzmann Solver (APBS) plugin in PyMOL (Schrödinger, LLC), with default electrostatic parameters. Surface potentials were visualised using the standard red–white–blue colour gradient, representing negative, neutral and positive regions, respectively.

## Supplementary material

online supplementary material 1.

## Data Availability

Coordinates for Alphafold models of CypD:C1qBP complex and C1qBP:Ca^2+^ interactions are deposited in ModelArchive Database with, respectively, accession code ma-0taq4 (password: fy0r32B9uz) and ma-4mxcf (password: 0FE2npnZET). Data generated and analysed for this study are included in this article (and its Supplementary).

## Open Access

This article has been published open access under our Subscribe to Open programme, made possible through the support of our subscribing institutions, learn more here: https://portlandpress.com/pages/open_access_options_and_prices#conditional

## Abbreviations

C1qBP: complement 1q binding protein
CsA: cyclosporin A
CypD: cyclophilin D
DLS: dynamic light scattering
DSF: differential scanning fluorimetry
HSQC: heteronuclear single-quantum coherence
ITC: isothermal titration calorimetry
NMR: nuclear magnetic resonance
PPIase: peptidyl prolyl cis-trans isomerase
SEC-MALS: size-exclusion chromatography-multi-angle light scattering
SLS: static light scattering
TMW: theoretical molecular weight
dRI: differential refractive index
mPTP: mitochondrial permeability transition pore
