# Supplementary material for: Cyclophilin D reduces Ca2+ sequestration by complement 1q binding protein
Source: Biochem J. 2025 Dec 17;482(24):1919–34. doi: 10.1042/BCJ20253361 (PMC12751049; doi:10.1042/BCJ20253361)
Supplement: online supplementary material 1. [file bcj-482-24-BCJ20253361-s001.docx]

**Cyclophilin D reduces Ca^2+^ Sequestration by Complement 1q Binding Protein**

Oluwatobi Adegbite^1,2,^ Yetunde Adegbite^1,3^, Catrin Pickering^1,4^, David N Criddle^1^, Lu-Yun Lian^1^

1. Institute of Systems and Molecular Biology, Biosciences Building University of Liverpool Liverpool L69 7BE, UK
2. Department of Biochemistry, Faculty of Basic and Applied Sciences, Osun State University, Osogbo, Osun State, Nigeria
3. Present Address: BioGrad Education, 61 Stephenson way, Wavertree Science Park, Liverpool L13 1HN, UK
4. Present Address: Department of Biology, Wentworth Way, University of York, York YO10 5DD, UK

**Materials and Methods**

**Supplementary 1**

**Cloning Primers for C1qBP into pETM11 vector**

C1qBP Forward primer: TCAGGGCGCCATGCACACCGACGGAGACAAAGC

C1qBP Reverse primer: CGGATCCCTATCACTGGCTCTTGACAAAACTCTTGAG

pETM11 backbone reverse primer: CGGTGTGCATGGCGCCCTGAAAATAAAGATTC

pETM11 backbone forward primer: GAGCCAGTGATAGGGATCCGAATTCGAGCTCCGT

The DNA sequence of the C1qBP insert (positions 238–867 bp) is provided below:

1 caccgctgcg gcgcgggtga tgccggcacg atgcgtccgg cgtagaggat cgagatctcg

61 atcccgcgaa attaatacga ctcactatag gggaattgtg agcggataac aattcccctc

121 tagaaataat tttgtttaac tttaagaagg agatatacca tgaaacatca ccatcaccat

181 caccccatga gcgattacga catccccact actgagaatc tttattttca gggcgccatg

241 cacaccgacg gagacaaagc ttttgttgat ttcctgagtg atgaaattaa ggaggaaaga

301 aaaattcaga agcataaaac cctccctaag atgtctggag gttgggagct ggaactgaat

361 gggacagaag cgaaattagt gcggaaagtt gccggggaaa aaatcacggt cactttcaac

421 attaacaaca gcatcccacc aacatttgat ggtgaggagg aaccctcgca agggcagaag

481 gttgaagaac aggagcctga actgacatca actcccaatt tcgtggttga agttataaag

541 aatgatgatg gcaagaaggc ccttgtgttg gactgtcatt atccagagga tgaggttgga

601 caagaagacg aggctgagag tgacatcttc tctatcaggg aagttagctt tcagtccact

661 ggcgagtctg aatggaagga tactaattat acactcaaca cagattcctt ggactgggcc

721 ttatatgacc acctaatgga tttccttgcc gaccgagggg tggacaacac ttttgcagat

781 gagctggtgg agctcagcac agccctggag caccaggagt acattacttt tcttgaagac

841 ctcaagagtt ttgtcaagag ccagtgatag ggatccgaat tcgagctccg tcgacaagct

901 tgcggccgca ctcgagcacc accaccacca ccactgagat ccggctgcta acaaagcccg

961 aaaggaagct gagttggctg ctgccaccgc tgagcaataa ctagcataac cccttggggc

1021 ctctaaacgg gtcttgaggg gttttttgct gaaaggagga actatatccg gattggcgaa

1081 tgggacgcgc cctgtagcgg cgcattaagc gcggcgggtg tggtggttac gcgcagcgtg

1141 accgctacac ttggcagcgc cctagcgccc gctcctttcg ctttcttccc ttccttttcc

1201 gccacgtttc gccgggtttt cccggta

**C1qBP Sequence**: MKHHHHHHPMSDYDIPTTENLYFQGAMHTDGDKAFVDFLSDEIKEERKIQKHKTLPKMSGGWELELNGTEAKLVRKVAGEKITVTFNINNSIPPTFDGEEEPSQGQKVEEQEPELTSTPNFVVEVIKNDDGKKALVLDCHYPEDEVGQEDEAESDIFSIREVSFQSTGESEWKDTNYTLNTDSLDWALYDHLMDFLADRGVDNTFADELVELSTALEHQEYITFLEDLKSFVKSQ*

**Supplementary 2**

**CypD R55K Mutation Sequencing**

The Translated region in the DNA sequence is from position 22 to 672

1 actttaagaa ggagatatac catgggcagc agccatcatc atcatcatca cagcagcggc 61 ctggtgccgc gcggcagcca tatggctagc atgactggtg gacagcaaat gggtcgggat 121 gaaaacctgt attttcaggg catgggtcgg gatgaaaacc tgtattttca gggcatgggg 181 aacccgctcg tgtacctgga cgtggacgcc aacgggaagc cgctcggccg cgtggtgctg 241 gagctgaagg cagatgtcgt cccaaagaca gctgagaact tcagagccct gtgcactggt 301 gagaagggct tcggctacaa aggctccacc ttccacaagg tgatcccttc cttcatgtgc 361 caggcgggcg acttcaccaa ccacaatggc acaggcggga agtccatcta cggaagccgc 421 tttcctgacg agaactttac actgaagcac gtggggccag gtgtcctgtc catggctaat 481 gctggtccta acaccaacgg ctcccagttc ttcatctgca ccataaagac agactggttg 541 gatggcaagc atgttgtgtt cggtcacgtc aaagagggca tggacgtcgt gaagaaaata 601 gaatctttcg gctctaagag tgggaggaca tccaagaaga ttgtcatcac agactgtggc 661 cagttgagct aactcgagca ccaccaccac caccactgag atccggctgc taacaaagcc

721 cgaaaggaag ctgagttggc tgctgccacc gctgagcaat aactagcata accccttggg 781 gcctctaaac gggtcttgag gggttttttg ctgaaaggag gaactatatc cggattggcg 841 aatgggacgc gccctgtagc ggcgcattaa gcgcggcggg tgtggtggtt acgcgcagcg 901 tgaccgctac acttgccagc gccctagcgc ccgctccttt cgctttcttc ccttcctttc 961 tcgccncgtt cgccggcttt ccccgtcaag ctctaaatcg ggggctccct tt

Translation

M G S S H H H H H H S S G L V P R G S H M A S M T G G Q Q M G R D E N L Y F Q G M G R D E N L Y F Q G M G N P L V Y L D V D A N G K P L G R V V L E L K A D V V P K T A E N F R A L C T G E K G F G Y K G S T F H K V I P S F M C Q A G D F T N H N G T G G K S I Y G S R F P D E N F T L K H V G P G V L S M A N A G P N T N G S Q F F I C T I K T D W L D G K H V V F G H V K E G M D V V K K I E S F G S K S G R T S K K I V I T D C G Q L S*

**Supplementary 3**

**CypD R82K Mutation Sequencing**

The Translated region in the DNA sequence is from position 24 to 674

1 taactttaag aaggagatat accatgggca gcagccatca tcatcatcat cacagcagcg 61 gcctggtgcc gcgcggcagc catatggcta gcatgactgg tggacagcaa atgggtcggg 121 atgaaaacct gtattttcag ggcatgggtc gggatgaaaa cctgtatttt cagggcatgg 181 ggaacccgct cgtgtacctg gacgtggacg ccaacgggaa gccgctcggc cgcgtggtgc 241 tggagctgaa ggcagatgtc gtcccaaaga cagctgagaa cttcagagcc ctgtgcactg 301 gtgagaaggg cttcggctac aaaggctcca ccttccacag ggtgatccct tccttcatgt 361 gccaggcggg cgacttcacc aaccacaatg gcacaggcgg gaagtccatc tacggaagca 421 agtttcctga cgagaacttt acactgaagc acgtggggcc aggtgtcctg tccatggcta 481 atgctggtcc taacaccaac ggctcccagt tcttcatctg caccataaag acagactggt 541 tggatggcaa gcatgttgtg ttcggtcacg tcaaagaggg catggacgtc gtgaagaaaa 601 tagaatcttt cggctctaag agtgggagga catccaagaa gattgtcatc acagactgtg 661 gccagttgag ctaactcgag caccaccacc accaccactg agatccggct gctaacaaag 721 cccgaaagga agctgagttg gctgctgcca ccgctgagca ataactagca taaccccttg 781 gggcctctaa acgggtcttg aggggttttt tgctgaaagg aggaactata tccggattgg 841 cgaatgggac gcgccctgta gcggcgcatt aagcgcggcg ggtgtggtgg ttacgcgcag 901 cgtgaccgct acacttgcca gcgccctagc gcccgctcct ttcgctttct tcccttcctt 961 tctcgccacg ttcgccggct ttccccgtca agctctaaat cgggggctcc ctttagggtt 1021 ccgatt

Translation

M G S S H H H H H H S S G L V P R G S H M A S M T G G Q Q M G R D E N L Y F Q G M G R D E N L Y F Q G M G N P L V Y L D V D A N G K P L G R V V L E L K A D V V P K T A E N F R A L C T G E K G F G Y K G S T F H R V I P S F M C Q A G D F T N H N G T G G K S I Y G S K F P D E N F T L K H V G P G V L S M A N A G P N T N G S Q F F I C T I K T D W L D G K H V V F G H V K E G M D V V K K I E S F G S K S G R T S K K I V I T D C G Q L S*

**Supplementary 4**

**Verification of C1qBP expression by western blot.**


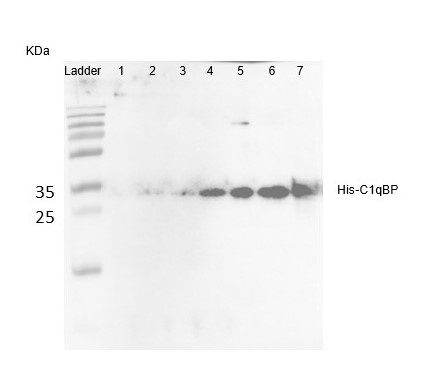


**Fig S4.** Representative Western blot showing a dilution series of recombinant His-tagged C1qBP. Lanes 1–7 correspond to increasing amounts of C1qBP protein, ranging from 0.5 ng to 32 ng in a twofold serial dilution. Detection was performed using a rabbit anti-C1qBP antibody (Proteintech, USA, Cat. No. 24474-1-AP).

**Supplementary 5**

**
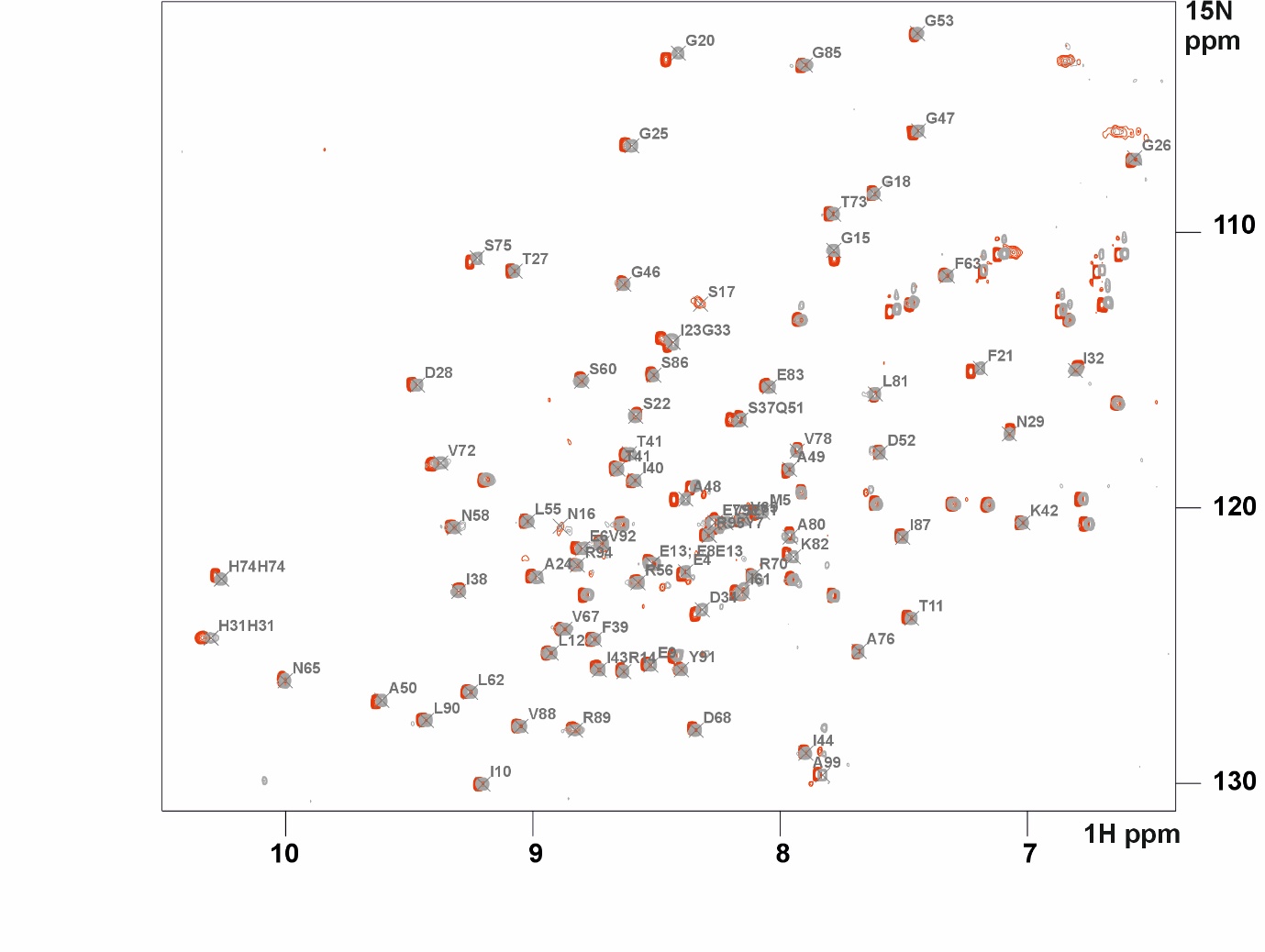
**

**Fig S5.** Two-dimensional ^15^N-^1^H HSQC spectrum (298K) of 50uM ^15^N uniformly labelled PSD95 PDZ1 alone (grey) and in the presence of 25uM C1qBP (red), pH6.5, 298K.

**Supplementary 6**

**
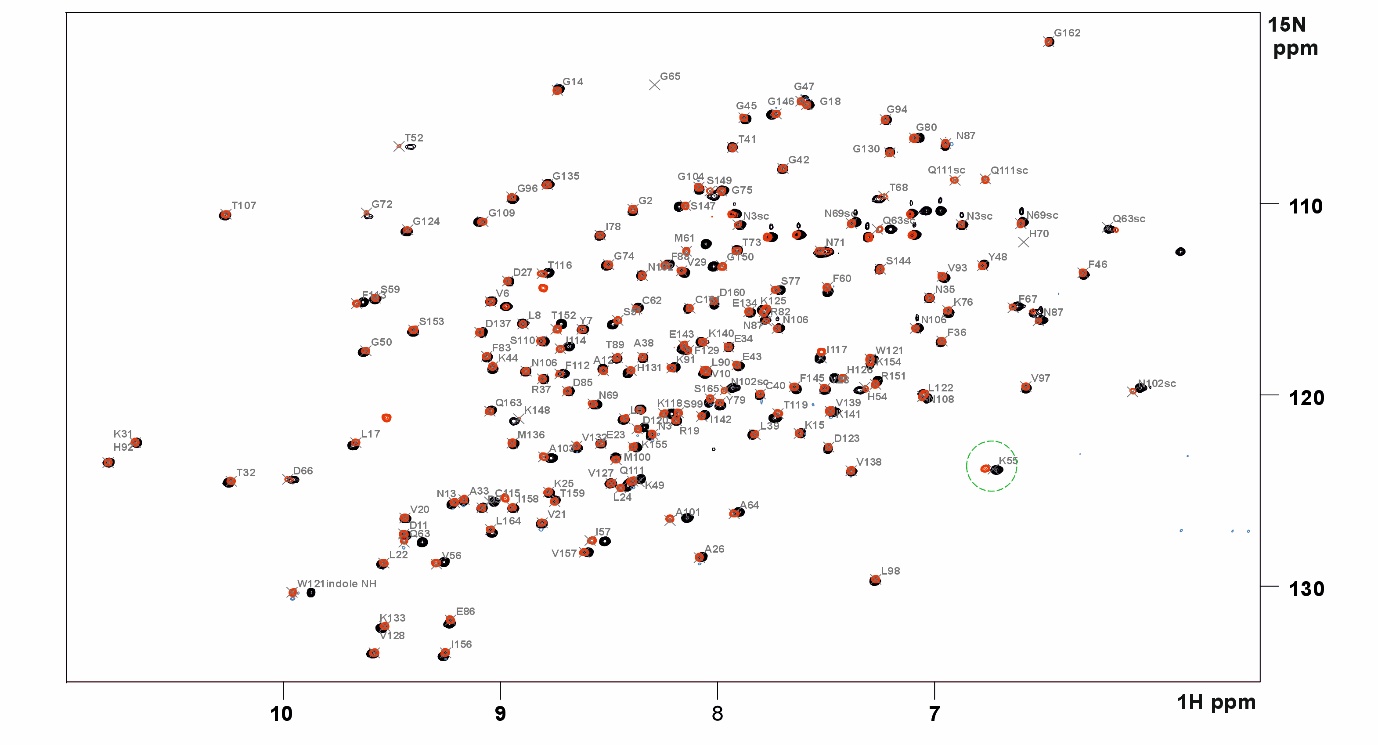
**

**Fig. S6** Two-dimensional ^15^N-^1^H HSQC spectrum (298K) of ^15^N uniformly labelled CypD R55K (red) overlay with wildtype CypD (black), 50uM protein, pH6.5, 298K. The assignment for K55 is indicated with green circle.

**Supplementary 7**

**
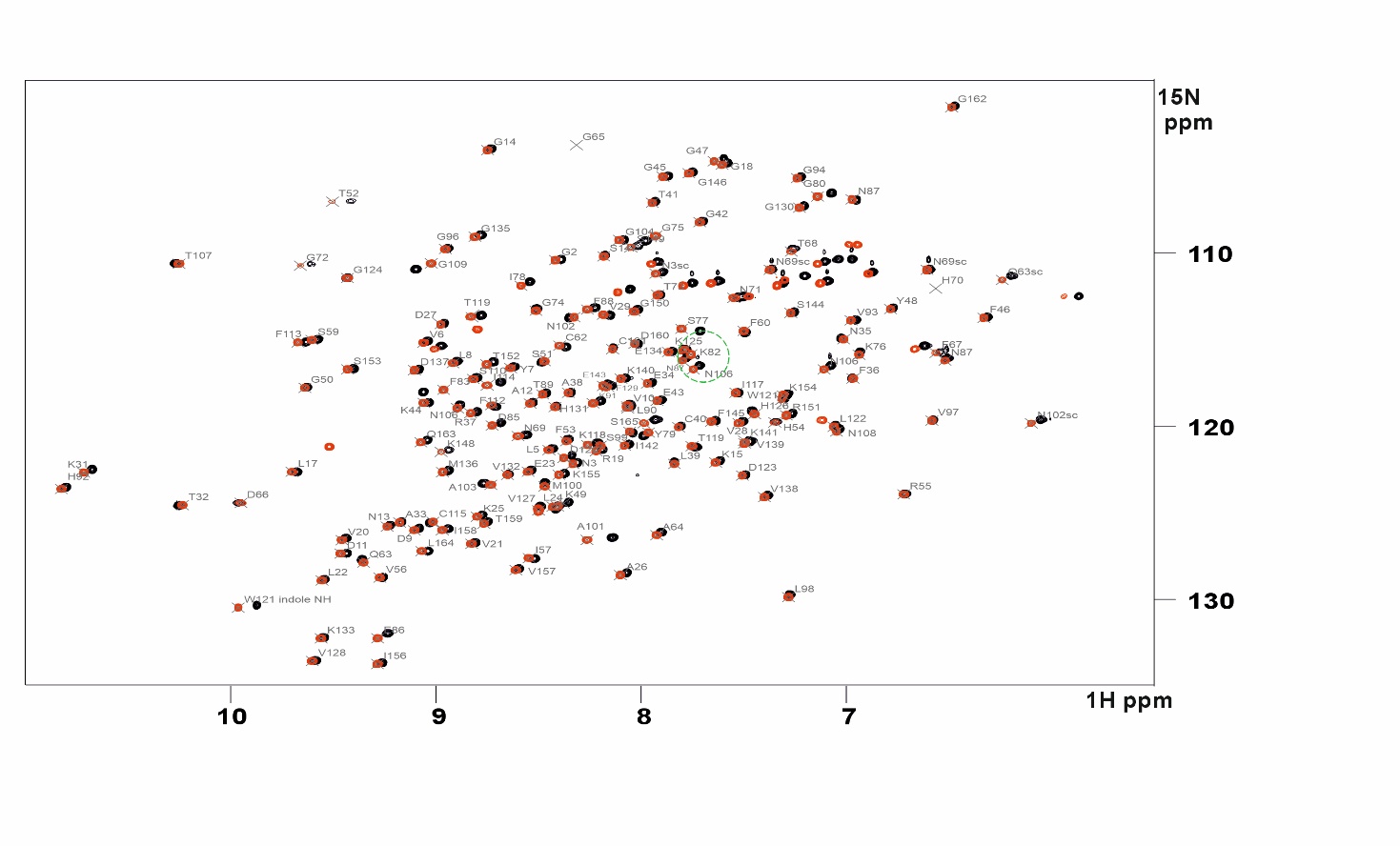
**

**Fig. S7** Two-dimensional ^15^N-^1^H HSQC spectrum (298K) of ^15^N uniformly labelled CypD R82K (red) overlay with wildtype CypD (black), 50uM protein, pH6.5, 298K. The assignment for K82 is indicated with green circle.

**Supplementary 8**

**ITC of CypD and Ca^2+^**

**Fig S8.** Isothermal titration calorimetry of CypD and Ca^2+^ at pH 7.4. No binding was observed. Need to add NMR data for CypD and Ca2+

**Supplementary 9**

**ITC of PDZ1 and Ca^2+^**

**Fig S9.** Isothermal titration calorimetry of PDZ1 and Ca^2+^ at pH 7.4. No binding was observed.

**Supplementary 10**

**AlphaFold model of mature C1qBP**


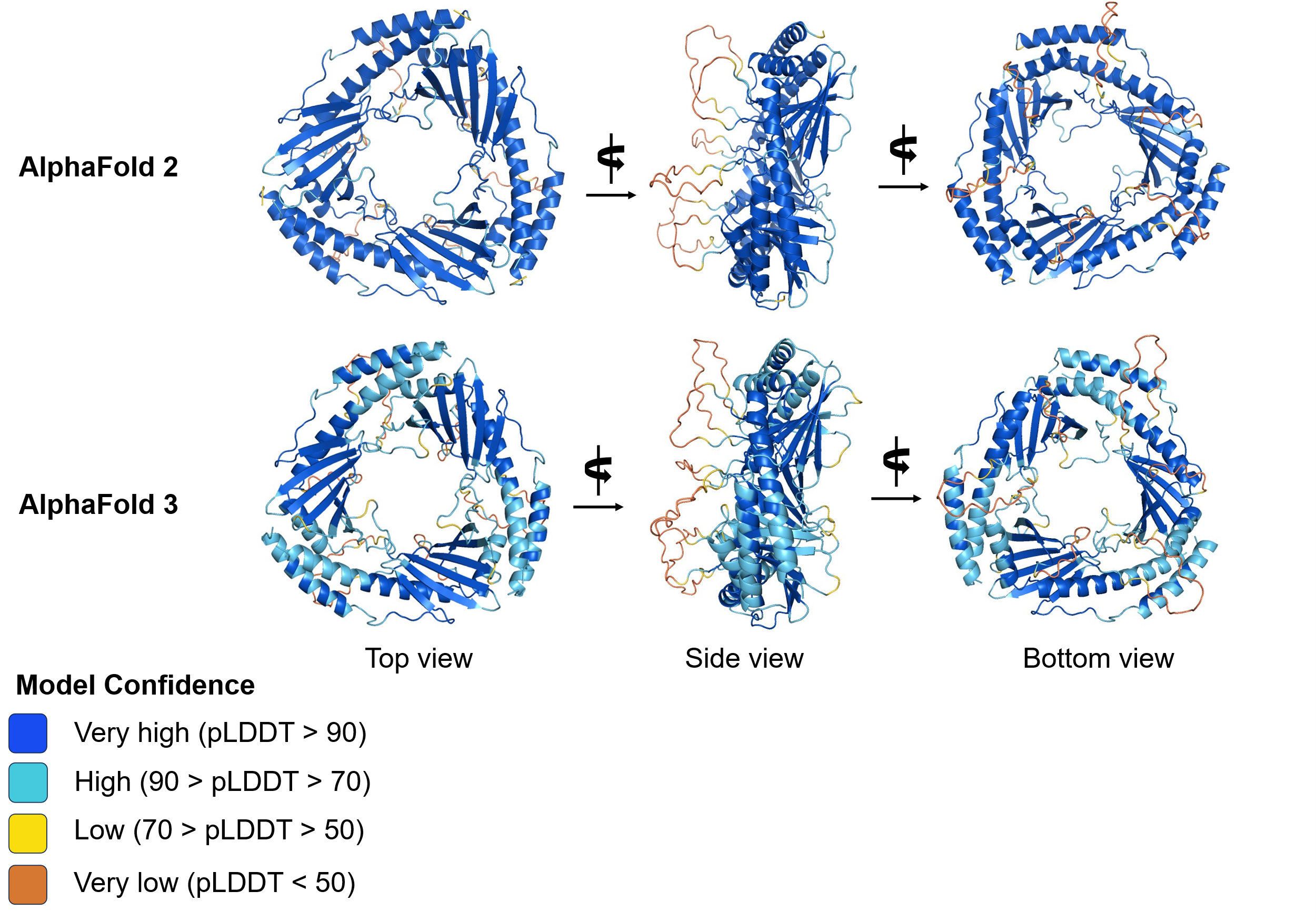


**Figure S10.** Predicted structural models of human C1qBP generated using AlphaFold 2 and AlphaFold 3. C1qBP structures predicted by AlphaFold 2 (top row) and AlphaFold 3 (bottom row) are shown in three orientations: top view (left), side view (middle), and bottom view (right). Protein models are coloured according to model confidence scores (pLDDT): very high (dark blue, >90), high (cyan, 70–90), low (yellow, 50–70), and very low (orange, <50). Both prediction algorithms reveal the trimeric ring-like organization of C1qBP, with overall high confidence in structured regions and lower confidence in intrinsically disordered regions.
